# Faecal Haemoglobin Estimated by Faecal Immunochemical Tests—An Indicator of Systemic Inflammation with Real Clinical Potential

**DOI:** 10.3390/diagnostics11112093

**Published:** 2021-11-12

**Authors:** Karen N. Barnett, Gavin R. C. Clark, Robert J. C. Steele, Callum G. Fraser

**Affiliations:** 1 Centre for Research into Cancer Prevention and Screening, University of Dundee School of Medicine, Dundee DD1 9SY, Scotland, UK; k.z.barnett@dundee.ac.uk (K.N.B.); r.j.c.steele@dundee.ac.uk (R.J.C.S.); 2 Public Health Scotland, Edinburgh EH12 9EB, Scotland, UK; gavin.clark@phs.scot

**Keywords:** chronic disease, data linkage, faecal haemoglobin, faecal immunochemical test, inflammation, multimorbidity, screening

## Abstract

Multimorbidity is the major cause of ill-health and premature death in developed countries. The ability to identify individuals at risk of developing chronic disease, particularly multimorbidity, reliably, and simply, and to identify undiagnosed disorders, is vital to reducing the global burden of disease. This narrative review, the first of recent studies, demonstrates that raised faecal haemoglobin concentration (f-Hb) is associated with increased all-cause and cause-specific mortality and with longer-term conditions including diabetes, hypertension, cardiovascular disease, and psoriasis, and with probable intake of particulate matter. We and others have hypothesized that elevated f-Hb (measured using a faecal immunochemical test) has considerable potential to identify individuals at risk of, or who already have, early stage, undiagnosed chronic disease. If f-Hb does prove to be an effective biomarker for chronic disease and multimorbidity, individuals with detectable f-Hb, but without an obvious source of gastrointestinal blood loss, could benefit from further assessment and early intervention. To test this hypothesis rigorously, longitudinal data-linkage methodology is required linking colorectal cancer screening data, and data on patients presenting with lower gastrointestinal symptoms, with routinely collected health information.

## 1. Introduction

Non-communicable disease is now the major cause of morbidity and premature death in developed countries, and chronic conditions frequently co-exist [1]. This has led to an increasing global burden of multimorbidity, defined as two or more chronic conditions in the same individual [2], particularly in the ageing population [3]. Multimorbidity not only leads to poorer quality of life [4] and increases the risk of death [5], but it also has very significant workforce and other resource implications for health care systems [6]. The ability to identify individuals at risk of developing multimorbidity, reliably and simply, efficiently, and effectively, and to identify undiagnosed disorders, is vital to reducing the global burden of disease. This ideal does not exist at present.

As discussed in detail in a recent comprehensive review [7], many developed countries now have colorectal cancer (CRC) screening programs. High numbers of ostensibly asymptomatic individuals are now undertaking quantitative faecal immunochemical tests (FIT) for haemoglobin, which have replaced the now considered obsolete [8], qualitative guaiac faecal occult blood test (gFOBT) in such programmes; the many advantages of FIT over gFOBT have been very well documented [7,9,10]. Moreover, quantitative FIT estimations are now widely used, especially in the UK and Spain [7], in the objective triage to bowel visualization of patients presenting in primary (and to a lesser extent, secondary) care with lower bowel symptoms [7,11]. Furthermore, the use of FIT in surveillance programmes following polypectomy is beginning to be examined in detail, in part due to the limited colonoscopy capacity in many countries, but also related to the need for better clinical care for such patients and the limited benefits of current endoscopic surveillance programmes [7].

A still unresolved conundrum is why a substantial proportion of those with faecal haemoglobin concentration (f-Hb) above the thresholds used in screening programmes and in the assessment of patients with symptoms, have no discernible colorectal pathology detected on colonoscopy [12]. Such test results are often termed “false positive results”. While there is considerable evidence of increasing future risk of colorectal neoplasia with increasing f-Hb [13,14,15,16,17], which holds for those both above and below the threshold for referral (i.e., f-Hb above the analytical limit of detection [18]), it is still only a small proportion of those with elevated f-Hb who go on to a diagnosis of colorectal neoplasia. This prompts the question of the source of bleeding in the remaining patients.

However, there is an ever growing literature evaluating the association between f-Hb and non-communicable diseases unrelated to CRC. This paper aims to briefly review these contributions, as shown in Table 1, in this first narrative summary on this topic, the studies having been published in a variety of specialist clinical journals. Moreover, here we also aim to put forward the unifying hypothesis for all of these findings in which it is proposed that elevated f-Hb has the potential to identify individuals at risk of, or who have, early stage undiagnosed chronic disease, and to provide an effective and efficient biomarker for multimorbidity.

## 2. Elevated f-Hb in Individuals with No Discernable Colorectal Pathology: A Review

### 2.1. f-Hb and Gastrointestinal Bleeding Caused by Drug Use

Elevated f-Hb in individuals with no pathological findings on colonoscopy is often attributed to the use of drugs that have the potential to cause gastrointestinal bleeding, but this remains controversial. A recent systematic review and meta-analysis has documented that FIT accuracy in CRC screening is not affected by the use of oral anti-coagulants, aspirin, and non-steroidal anti-inflammatory drugs [29]. In contrast, another recent review suggested that aspirin, antiplatelet agents, and oral anti-coagulants significantly lowered the positive predictive value (PPV) of FIT for detecting advanced colorectal neoplasia (ACRN) and thus might increase the number of false positive FIT results [30]. Recent interest has been focused on f-Hb and the use of proton pump inhibitors (PPI) with the suggestion [31] that PPI could cause false positive FIT results, supporting earlier work [32,33]: however, this finding has been refuted [34]. The mechanism by which PPI resulted in an increase in the false positive rate was stated to be unknown, but the hypothesis presented in [31] was that, consistent with the finding that antibiotic therapy was also associated with false positive results, it could be inferred that gut dysbiosis induced by PPI could be the cause. However, the new hypothesis advocated here below provides an alternative and possibly more attractive explanation. It is considered that such results could arise because individuals who are taking drugs purportedly causing elevated f-Hb are, by definition, at high risk of having or developing chronic conditions (see Section 2.3). There is now good evidence, discussed below, that elevated f-Hb is a marker of susceptibility to such conditions, probably acting as an index of systemic inflammation, so that the drugs may not be causing the raised f-Hb, but merely acting as a parallel marker.

### 2.2. f-Hb and All-Cause Death and Chronic Disease

Observations from a well-established CRC screening programme in Taiwan demonstrated that, in screened individuals, increasing f-Hb measured by quantitative FIT was associated with an increasing risk of death not only from CRC, but also from all causes [19]. It was suggested that one biological explanation for this effect might be that an increase in f-Hb is associated with a decrease in systemic blood haemoglobin concentration, which had been demonstrated in a previous study to be a strong predictor of all-cause death [35]. Another possible explanation offered was that an increase in f-Hb reflected systemic inflammation. The adjusted hazard ratios (HR) increased from 1.43 (95% CI: 1.08–1.88) for baseline f-Hb of 20–39 ng/mL, to 3.41 (95% CI: 2.02–57·5) for a baseline concentration of 80–99 ng/mL (trend test *p* < 0·0001), relative to 1–19 ng/mL. Those not referred for further investigation had the highest risk of incident colorectal neoplasia with adjusted HR 8.46 (95% CI: 6.08–11.76). However, the precise non-CRC causes of death were not investigated, and corrections for sex, age, and deprivation, all of which are correlated with f-Hb (and also linked to multimorbidity) [36], were not made.

A study performed in Scotland demonstrated that an abnormal (positive) guaiac faecal occult blood test (gFOBT) result, which can be considered as a marker for elevated f-Hb, was shown to be associated with an increased risk of death, not only from CRC, but also from all-causes, cardiovascular disease, respiratory disease, digestive disease, neuropsychological conditions, and endocrine disease [20]. After correcting for sex, age, deprivation, and medicines that might cause gastrointestinal bleeding, these associations still held. For example, those with a positive test result had a higher risk of dying than those with a negative result from CRC: HR 7.79 (95% CI: 6.13 to 9.89), *p* < 0.0001, and from all non-CRC causes: HR 1.58 (95% CI: 1.45 to 1.73), *p* < 0·0001. The hypothesis proposed was that a systemic inflammatory state may be exhibited by subclinical colonic inflammation, undetected on colonoscopy, but with occult bleeding as a result. The normal colon is recognised to contain inflammatory cells in the submucosa, reflecting its ongoing requirement to eliminate organisms that breach the epithelium [37]. It was proposed that f-Hb might have potential as a modifiable biomarker that could be used to assess the value of lifestyle and prescribe interventions with the aim of reducing the risk of premature mortality. In addition, for the different existing patterns of mortality in populations across the world, f-Hb might also be of value in investigating the reason for these dissimilarities.

The association between high f-Hb and cardiovascular disease has also been demonstrated in a large community-based FIT screening programme in Taiwan in which 33,355 healthy individuals, free from cardiovascular disease, were followed up. A positive relationship between elevated f-Hb and the development of cardiovascular disease and subsequent cardiovascular death was reported [21]. The risk of cardiovascular disease increased with baseline f-Hb, showing a significant elevated risk of cardiovascular disease in parallel with the incremental f-Hb with adjusted HR of 1.04, 1.10, 1.40, and 1.23 for f-Hb of 1–19, 20–49, 50–99 and ≥100 ng/mL, trend test, *p* < 0.0001, compared with the reference group with undetectable f-Hb. Additionally highlighted was the need for a better understanding of the biological mechanisms underlying the relationship between f-Hb and cardiovascular disease, and the potential use of FIT in a primary prevention strategy was mentioned. Interestingly, the prediction ability of the model additionally including f-Hb for the risk of cardiovascular diseases was also higher than that of the model including traditional atherosclerotic risk factors.

Similarly, researchers in South Korea have highlighted the broader clinical utility of FIT, based on data from a nationwide population cohort study of over six million individuals. Results from a multivariable analysis, adjusting for age, sex, smoking, alcohol consumption, regular exercise, diabetes mellitus, hypertension, dyslipidaemia, and body mass index found an increased risk of ischemic stroke, myocardial infarction (MI), and all-cause mortality among those with f-Hb above the threshold used in the screening programme [22]. The risk of ischemic stroke was higher in the test result positive population with adjusted HR of 1.09; 95% CI: 1.07–1.11). Similarly, those with positive test results were at an increased risk of MI (adjusted HR, 1.09; 95% CI: 1.06–1.12). Moreover, increased all-cause mortality was observed in the positive test result population (adjusted HR, 1.15; 95% CI: 1.07–1.23). It was postulated that estimation of occult blood in faeces may provide useful information in addition to its conventional role in CRC screening.

Furthermore, a Japanese study has demonstrated an association between FIT positivity and glycated haemoglobin (HbA1c) concentrations, as used in the diagnosis and monitoring of diabetes mellitus, in an apparently healthy population undergoing an annual health check. Multivariate logistic regression analysis showed that, compared with HbA1c of ≤5.69%, HbA1c of ≥6.5% was significantly associated with positive test results. The association remained when adjusting for demographic and lifestyle factors [23].

This relationship was strongly supported in a recent South Korean study 24]. During a mean follow-up of 6.5 years, the incidence rates of diabetes were 11.97, 13.60, 14.53, and 16.82 per 1000 person years in the FIT negative, one-positive, two-positive, and three-positive groups, respectively. The HR for the incidence of diabetes was 1.14 (95% CI: 1.12 to 1.16; HR, 1.21; 95% CI: 1.16 to 1.27; and HR, 1.40; 95% CI: 1.28 to 1.55) in the one-positive, two-positive, and three-positive test result groups compared with the negative test result group, respectively. It was concluded that positive FIT results were associated with a significantly higher risk of diabetes, suggesting that the FIT can be of value, not only in CRC screening, but also as a surrogate marker of systemic inflammation.

Recently, a South Korean study aimed to determine whether positive FIT results are associated with death from various causes in the population [25]. Data were collected from screening participants who underwent FIT. FIT positive (i.e., f-Hb above the set threshold) participants had a higher mortality rate than FIT negative (i.e., f-Hb below the set threshold) participants from CRC. Despite adjusting for age, sex, smoking status, alcohol consumption habits, body mass index, comorbidity, and aspirin use, FIT positivity was associated with an increased risk of dying from all non-CRC causes, (adjusted HR, 1.17; 95% CI: 1.15 to 1.18) and CRC (adjusted HR, 5.61; 95% CI: 5.40 to 5.84). Additionally, FIT positivity was significantly associated with increased mortality from circulatory disease (adjusted HR, 1.14; 95% CI: 1.11 to 1.17), respiratory disease (adjusted HR, 1.14; 95% CI: 1.09 to 1.19), digestive disease (adjusted HR, 1.57; 95% CI: 1.48 to 1.66), neuropsychological disease (adjusted HR, 1.08; 95% CI: 1.01 to 1.16), blood and endocrine diseases (adjusted HR, 1.10; 95% CI: 1.04 to 1.17), and external factors (adjusted HR, 1.16; 95% CI: 1.11 to 1.20). It was concluded that positive FIT results (i.e., elevated f-Hb) are associated with an increased risk of mortality from CRC and, much more importantly, for various other chronic diseases, suggesting that it could be a predictor of mortality independent of its association with CRC.

### 2.3. f-Hb and Other Inflammatory States

In Scotland, to further explore the relationship of f-Hb with chronic disease, the associations between f-Hb and prescription of medicines for a variety of chronic conditions were investigated [26]. In a cross-sectional approach, data on participants in the gFOBT-based Scottish Bowel Screening Programme were studied by linking the individuals’ gFOBT result (classed as abnormal or normal: i.e., elevated or usual f-Hb) with prescribing of medicines data when the gFOBT was undertaken. Participants with an abnormal gFOBT result (i.e., an elevated f-Hb) were more likely to have been prescribed medicines for heart disease, hypertension, diabetes, and depression than those with a normal test result. After adjustment for sex, age, and deprivation, the associations were maintained with OR 1.35 (95% CI: 1.23 to 1.48), 1.39 (95% CI: 1.27 to 1.52), 1.35 (95% CI:1.15 to 1.58), and 1.36 (95% CI: 1.16 to 1.59), all *p* < 0.0001 for the four medicine categories, respectively). It was thought that these results provided additional and substantial weight to the concept that detectable f-Hb is associated with a range of common chronic conditions that have a component of systemic inflammation. In addition, it was considered that f-Hb might have potential in identifying those individuals who are at high risk of developing chronic conditions or who are at an early stage of disease.

In another recent South Korean study involving screening participants, positive FIT results were associated with psoriasis, a chronic inflammatory skin disease, suggesting to the authors a relationship between skin disorders and gut health [27]. The incidence of psoriasis (per 1000 person-years) was 3.76 versus 4.14 (FIT negative test result versus FIT positive test result group) during a median follow-up of 6.68 years. In the multivariable-adjusted model, the HR for psoriasis was 1.03 for one-positive result, 1.12 for two-positive results, and 1.34 for three-positive FIT results compared with the negative test results. Moreover, it was documented that epidemiological studies have shown that psoriasis is associated with dyslipidaemia, obesity, hypertension, diabetes mellitus, and metabolic syndrome, all of which, as documented above, are associated with elevated f-Hb in the absence of obvious colorectal pathology. After adjusting for these confounding factors, positive FIT results were associated with a proportionally increased risk of psoriasis. It was admitted that the mechanism underlying the association between f-Hb and the risk of psoriasis was unclear, but it was pointed out that there are several possibilities. A chronic inflammatory environment may link positive FIT results and psoriasis: FIT can detect inflammatory conditions manifesting as mucosal ulceration and occult blood loss as shown in studies on f-Hb and mucosal healing in inflammatory bowel disease [38]. A colonic inflammatory status and the consequent disruption of the intestinal epithelial barrier (i.e., “leaky gut”) may lead to detectable f-Hb. Indeed, there is evidence that systemic inflammation can be triggered by a leaky gut [39]. Recently, a range of biomarkers linking a leaky gut and subsequent bacterial translocation to metabolic health indices have been identified [40]. Zonulin was the most strongly associated with metabolic health markers and serum C-reactive protein (CRP), which is applied in routine care as a chronic inflammation marker. It would be very interesting to explore whether positive relationships exist between f-Hb and zonulin and other biomarkers in faeces, and perhaps in blood, and whether these biomarkers could be estimated concomitantly using the specimens collected for f-Hb estimation in the collection devices used with modern FIT analytical systems, as has been performed with studies on the faecal microbiome [41].

Finally, an association of ambient fine particulate matter (PM2.5) in the air with elevated f-Hb has been demonstrated [28]. With data from a large scale FIT based screening program, and with nationwide exposure data available in Taiwan, the effect of PM.2.5 on a possible inflammatory response from cells lining the colorectal lining was investigated by using the proxy indicator of FIT positivity (i.e., elevated f-Hb). Individuals in the high PM2.5-exposure group showed higher FIT positivity (using 20 µg Hb/g faeces as the threshold) than those in the low exposure group (9.9% vs. 8.4%). Moreover, it was shown that there was a positive association between monthly averaged PM2.5 concentrations and FIT positivity and an increased risk of 11% (95% CI: 10–12). PM2.5 enhanced the risk of being in a preclinical state by 14% (95% CI: 10–18) and that of subsequent progression from a pre-clinical to a clinical state by 21% (95% CI: 14–28).

## 3. The Hypothesis

The above narrative review amply demonstrates that there is now convincing and ever-growing evidence to support the hypothesis that elevated f-Hb is associated with increased all-cause and cause-specific mortality and with long-term conditions including diabetes, hypertension, cardiovascular disease, psoriasis, and intake of particulate matter. To our knowledge, there are no works that suggest that f-Hb is not associated with the systemic inflammation of chronic conditions. The conditions studied to date are all associated with systemic inflammation, and it is likely that this is reflected in increased, albeit subclinical, colonic inflammation, which in turn is manifested in occult bleeding detected by FIT. This may also partly explain the associations seen between f-Hb and the detection of adenoma at colonoscopy, since adenoma are not necessarily bleeding when viewed endoscopically. It may be that the inflamed colon is more likely to lead to adenomas, which are detected by increased f-Hb, rather than bleeding directly from the adenoma. Thus, FIT might be an effective, simple, inexpensive, and reliable biomarker for systemic inflammation, and hence for most chronic non-communicable disease. It follows that individuals with detectable f-Hb, without an obvious source of gastrointestinal bleeding, might benefit from further assessment and early intervention. Since FIT-based CRC screening is now very widely undertaken in opportunistic and programmatic approaches, although individuals who participate in screening tend to be healthier than those who do not, many individuals without symptoms of disease will have had a detectable f-Hb estimate generated. This finding might be applied to communicate the risk of future chronic conditions to the individual as well as to suggest strategies for the minimization of risk. Anecdotally, in the United Kingdom, it is said that about 10% of consultations in primary care are with patients presenting with lower gastrointestinal symptoms; FIT is becoming widely used in the triage of such patients [7] and, as a consequence, a quantitative estimate of f-Hb is also available for a further large cohort of individuals. We suggest that interventions might be particularly directed to (1) those who participate in FIT-based screening and have a detectable f-Hb below the threshold used for referral for bowel visualization; (2) participants in such a screening who have a f-Hb above the threshold, but who have no detectable colorectal disease; and (3) those patients who present in primary or secondary care with symptoms, have a detectable f-Hb, and undergo further investigations, but have no significant bowel disease detected. Clearly, these suggestions are currently speculative and do require prospective studies before implementation can be instituted.

## 4. The Future

Longitudinal data linkage studies are well-suited to determine the extent to which f-Hb is associated with disease and multimorbidity and, crucially, how f-Hb might be associated with the onset and progression of chronic disease processes. The first stage in testing the hypothesis would be to link CRC screening test results (quantitative f-Hb) with other routinely collected health datasets that provide information on the incidence and prevalence of chronic disease. For example, in Scotland, f-Hb obtained within the Scottish Bowel Screening Programme could be linked with routine laboratory data, the Scottish Morbidity Records of General/Acute Inpatient and Day Case episodes, the Scottish Cancer Registry, General Register Office (death records), and Prescribing Information Scotland (to help identify chronic disease that has not resulted in a hospital episode). Similar local linkage studies could be performed for those patients with symptoms and elevated f-Hb but no obvious gastrointestinal pathology. Likewise, it might be that if FIT becomes more widely used in post-polypectomy surveillance, as described recently [42], it is certain that these patients with detectable f-Hb, but no pathology, would benefit from intervention.

Using advanced statistical approaches, this strategy could be used to determine the f-Hb threshold that represents a significant risk of chronic disease and multimorbidity. Once this has been established, prospective studies would be required to determine the clinical impact of using FIT to identify individuals who would benefit from early intervention or preventative measures to treat or arrest the development and progress of the common components of multimorbidity.

If f-Hb were to prove an effective and efficient biomarker for chronic disease, particularly multimorbidity, the potential impact would include a fundamental change to our current health care systems and how individuals currently present with chronic conditions. FIT could offer significant opportunities to reduce the burden of chronic disease and multimorbidity through initiating prompt interventions to prevent, or minimize, the impact of these disease processes. These could include drug therapy, weight management and exercise interventions, dietary (including alcohol) advice, and smoking cessation. Although there is now ample evidence that FIT appears very promising as an indicator of systemic inflammation, it must be realized that there are issues that must be considered before wide adoption of this application of f-Hb [43]. These include the difference between the relative risk and relative odds, the need to estimate the absolute risk reduction that might be gained through this application of f-Hb, the importance of carefully assessing the expected and the accepted false positive rate together with the detection rate, the PPV and its relationship with the prevalence of the disease, and the need for careful assessment of the benefits and risks and burdens such as the impact of this application on the provision of additional health care. Ultimately, after appropriate prospective research, it is possible that the efficacy of these interventions could be monitored by the objective analyses of serial estimates of f-Hb using FIT, although little is known at the present time about the utility of this strategy, particularly in symptomatic patients [44]: the use of serial f-Hb in monitoring responses to interventions in individuals is, as yet, speculative.

## Figures and Tables

**Table 1:** Studies demonstrating the association between faecal haemoglobin concentration and diseases other than colorectal neoplasia and inflammatory bowel disease.

First Author [Reference]	Country	Number of Subjects	Test for Occult Blood in Faeces ^1^	Association with Faecal Haemoglobin Concentration
Chen [19]	Taiwan	18,573	FIT	Risk for death from CRC ^2^ and all-cause death.
Libby [20]	Scotland	134,192	gFOBT	Increased risk of dying from circulatory disease, respiratory disease, digestive diseases (excluding CRC), neuropsychological disease, blood, and endocrine disease and non-colorectal cancers.
Chien [21]	Taiwan	33,355	FIT	Risk of cardiovascular diseases.
Moon [22]	South Korea	6,277,446	FIT	Ischemic stroke, myocardial infarction, and all-cause mortality.
Nakajima [23]	Japan	12,836	FIT	Glycated haemoglobin.
Kim [24]	South Korea	7,946,393	FIT	Significantly higher risk of diabetes.
Jung [25]	South Korea	5,932,544	FIT	Increased risk of mortality from circulatory disease, respiratory disease, digestive disease, neuropsychological disease, blood and endocrine diseases, and external factors.
Libby [26]	Scotland	134,192	gFOBT	More likely to have been being prescribed medicines for heart disease, hypertension, diabetes, and depression.
Lee [27]	South Korea	1,395,147	FIT	Risk of psoriasis.
Ku [28]	Taiwan	4,628,995	FIT	Ambient fine particulate matter (PM2.5).

^1^ FIT: faecal immunochemical test; gFOBT: guaiac faecal occult blood test. ^2^ CRC: colorectal cancer.

## Data Availability

We did not report any original date: this is a review and hypothesis paper.
